# ACHIEVING QUALITY PATIENT-CENTERED CARE IN THE NURSING HOME SETTING

**DOI:** 10.1093/geroni/igac059.228

**Published:** 2022-12-20

**Authors:** Colleen Galambos

**Affiliations:** University of Wisconsin-Milwaukee, Milwaukee, Wisconsin, United States

## Abstract

In 1987, The Nursing Home Reform Act was enacted as part of the Omnibus Reconciliation Act of 1987 (OBRA 87). At that time, the Health Care Finance Administration (now Centers for Medicare and Medicaid Services) issued comprehensive regulations and survey processes to “ensure that residents of nursing homes receive quality care that will result in their highest practicable physical, mental, and social well-being.” Despite this landmark legislation, nursing homes struggle to provide quality care, and are additionally challenged by natural disasters and pandemics. This presentation will report on recommendations that examine, structures, policies, and care models that promote change and innovation, with a focus on safety, environmental modifications, patient centered approaches, and quality care. Dr. Colleen Galambos, a professor at University of Wisconsin-Milwaukee, with expertise in nursing home care delivery and quality improvement will present the care delivery recommendations from the NASEM report.

